# The Influence of the Type of Dry-Cured Italian PDO Ham on Cathepsin B Activity Trend during Processing

**DOI:** 10.3390/foods10123123

**Published:** 2021-12-16

**Authors:** Edi Piasentier, Nicoletta Pizzutti, Giovanna Lippe

**Affiliations:** 1 Department of Agricultural, Food Environment and Animal Sciences, University of Udine, 33100 Udine, Italy; nicoletta.pizzutti@live.it; 2 Department of Medicine, University of Udine, 33100 Udine, Italy; giovanna.lippe@uniud.it

**Keywords:** proteolysis, cathepsin B, dry-cured ham, Italian PDOs

## Abstract

Cathepsin B activity was measured during processing in hams originating from the main Italian prosciutto PDOs: Parma, San Daniele and Toscano. Sixty-five heavy pig thighs, from sixty-five Italian large white x Italian Landrace pigs bred and slaughtered in the same conditions were considered. Five thighs represented the post-mortem control time. The other 60 were distributed one plant per PDO, following a balanced plan. The thighs were sampled at the *biceps femoris* in groups of four per plant in the following ripening phases: salting, resting, drying, greasing, end of curing. The activity of the Cathepsin B (U/g protein) was determined by means of fluorescence measurements. The Cathepsin B ripening trend of the various PDOs was significantly different, particularly during the initial and mid-curing stage. This activity correlates with the proteolysis index through a PDO dependent pattern, indicating that different processing conditions can influence the quality of prosciutto, since they determine its biochemical development.

## 1. Introduction

Almost 80% of pigs born and raised in Italy are destined for the market of traditional Italian dry-cured ham (‘Prosciutto’), the production of which is largely controlled by a protected designation of origin (PDO) to achieve products with reproducible, high-quality sensory characteristics. The most important PDOs for Italian dry-cured ham are: PDO Prosciutto di Parma (PA), PDO Prosciutto di San Daniele (SD), PDO Prosciutto Toscano (TO), which processed 7.8, 2.5 and 0.30 million conforming thighs, respectively, in 2020 [1].

The processing conditions of the three PDO hams differ. SD is the only one that retains the trotter. In the dressing phase, upon receipt of the thighs, TO is cut in a “V” shape by removing a greater part of the skin than the other two PDO types, exposing a larger portion of the *semimembranosus* muscle; this increases the penetration of salt into the ham. The addition of salt to the fresh thigh (moisture 79%, water activity, a_w_ 1; protein 15–23%; fat 2.5%; salt 1%) decreases the a_w_, ensuring the preservation of the dry-cured ham (moisture 57–60%, a_w_ 0.85, protein 29–31%, fat 3–4%, salt 5–6%) which, at the end of a maturation period lasting at least 12 months, is 34–36% of the initial weight of the green thigh [2]. The salt can be added to the three PDOs in different forms: rubbed in dry and wet for PA, dry and ad libitum for SD, and dry and mixed with pepper and vegetable flavorings for TO. The ‘ripasso’ i.e., the replacement of old salt with new during salting, is only performed once for PA and SD and twice for TO. Ultimately, according to the specifications of each PDO consortium (http://www.prosciuttodiparma.com/ (accessed on 14 November 2021); http://consorzio.prosciuttosandaniele.it/ (accessed on 14 November 2021); http://www.prosciuttotoscano.com/ (accessed on 14 November 2021)), the whole salting phase duration is as follows. For PA, about one week plus a further 15–18 days to obtain a second thin coating of salt. For SD, a number of days corresponding to the thigh’s weight in kilograms. For TO, about 3–4 weeks. In the pre-resting phase, SD is pressed into a typical guitar shape, facilitating the penetration of the salt. The subsequent phases of resting, rinsing, drying, and curing [3] are similar in the three PDOs except for the ‘sugnatura’ (greasing, a phase in mid-curing during which the exposed lean surface is covered with ‘sugna’, a mixture of flare fat and wheat or rice flour for drying control): for TO, the classic sugna is supplemented by pepper and vegetable flavorings.

The biochemical activities that occur during processing, which are dependent on both the protein fraction and the lipid component, form the basis of the physical and chemical changes described above, which in turn are responsible for the typical sensory properties of the ham [4,5]. In general, the proteins are degraded by endoproteases into shorter peptides, and these represent the substrate of the exopeptidases, which hydrolyze them into free amino acids. It is now widely accepted that during ham maturation, proteins are degraded by muscle proteolytic systems, which guarantees their physiological activation and turnover in vivo [6]. The systems are distinguished by their different intracellular locations and functional properties (substrate specificities, optimal pH, etc.): calpains, cysteine-endopeptidases located in the cytoplasm; proteasome, a multi-enzymatic complex with a high molecular weight, also located in the cytoplasm; and cathepsins, lysosomal proteases of the papain family [7]. The latter are divided into cysteine, serine, and aspartyl proteinases, and include both endo- and exo-peptidases activated by acid pH [8,9]. During post-mortem, the proteolytic activity affects both myofibril and sarcoplasmatic proteins. The contribution of calpains and the proteosome is limited, due to their low stability, while the role of cathepsins is considered fundamental, particularly B, D and L which, together with H, represent the most abundant lysosomal proteases in the muscle [10]. When released by the lysosomes, as occurs during post-mortem, these are controlled by a family of inhibitory proteins called cystatins [11,12], which thus perform an essential role in controlling proteolysis during the initial phases. In this period, cathepsin B appears to be particularly active in relation to desmin, myosin, actin and tropomyosin I [13]. The degradation of desmin is a critical phase during post-mortem, as the meat acquires its characteristic tenderness and starts to lose water. The action of cathepsin B on desmin is mediated by its exopeptidase activity, and by its dipeptidyl peptidase activity [14].

Cathepsin B is also fundamental to produce dry-cured ham and green thighs with high activity have been reported to yield more proteolyzed dry-cured hams [15,16,17]. In addition, cathepsin B is particularly stable compared with other cathepsins throughout ham processing [18]. In any case, the transformation of fresh thighs into dry-cured ham interferes with its normal enzymatic activity; indeed, various authors have noted that the salt concentration, temperature, water activity, and pH interfere with cathepsin B [2,15,17,18,19,20]. These different technological conditions and the consequent intensity of proteolysis produce specific sensory properties in the hams [21,22], whose molecular mechanisms have been studied thanks to proteomics approaches [23].

The purpose of this study is to investigate the influence of the production process on cathepsin B activity in industrial conditions, measuring its variation in different processing phases in hams from Parma and two other important Italian PDOs, San Daniele and Toscano, obtained from coetaneous and homogeneous pigs bred together. The relationship between the intensity of the proteolysis and the sensory properties of the various PDO hams is also discussed, utilizing the profile provided by trained assessors and the consumer preferences for the three PDO as evaluated in already published papers [21,22]. To our knowledge, this is the first time San Daniele and Toscano cathepsin B trend during seasoning has been examined and compared with Parma.

## 2. Materials and Methods

### 2.1. Thigh Origin

The test material comprised heavy pig thighs, with morphological characteristics suitable for the PDO chain, in line with the requirement of conformity with the specifications of the relevant PDO prosciutto. The thighs came from 66 pigs, crosses of large white x Landrace of Italian selection (ANAS), bred on a single farm with the same balanced diet and slaughtered in the same abattoir, in three batches of 22 pigs each, at 6 week intervals, as described elsewhere, together with carcass and meat attributes [22,23].

### 2.2. Plan of Distribution and Ripening of the Thighs

Two thighs per slaughter batch were randomly obtained to gather the initial control time 0 (T0), which ultimately included five thighs because one was accidentally lost. The other 60 were distributed to three prosciutto plants belonging to the three different PDOs (Parma, San Daniele, Toscano), in accordance with a balanced distribution plan. Each PDO thus received 20 thighs, in batches of 10 from two of the three slaughters performed in total. Upon entering the processing plant the weights were recorded after dressing (initial weight).

The processing program was monitored, and the days since receipt recorded in the following phases:

- T0, slaughter
- T1, out of salting (salting)
- T2, introduction to the resting room (resting)
- T3, after washing and drying (drying)
- T4, mid-curing, after greasing (greasing)
- T5, end of curing (curing)

Four thighs were sampled per prosciutto plant in each phase, apart from the first one described above. During maturation the weight of all the thighs still present was recorded in the following processing phases:

- out of salting
- pre- and post-trimming
- post-washing and drying
- pre- and post-sugnatura
- pre- and post-‘stuccatura’ (only for SD, a second greasing using ‘stucco’, a sugna mixture richer in flour)
- end of curing.

These data were used to calculate the weight losses net of trimming subtractions and additions of sugna and stucco, i.e., an estimate of the water losses on the specific day of ripening on which the individual thigh was sampled. The losses were expressed in g/kg of dressed-thigh (initial) weight.

### 2.3. Collection of the Sample for Analysis

A sample from the same location of the *biceps femoris* muscle of around 100 g was taken from the thigh by cutting a 5 cm-thick slice transversally from the thigh, at about 8 cm from the femoral head [21]. The sample was then divided into portions. The first portion was ground and vacuum-preserved at a temperature of −20 °C until the determination of the following:

- Moisture content [24];
- Sodium chloride content, using the Volhard method [24];
- a_w_ (water activity; AquaLab Dew Point Water Activity Meter 4TE, Decagon Devices, Inc., Pullman, WA, USA);
- Proteolysis index: the ratio between the Non Protein Nitrogen (NPN) and Total Nitrogen (TN); i.e., NPN/TN × 100. The proteins were precipitated using 20% (*w*/*v*) trichloroacetic acid and NPN and the TN contents were determined according to the Kjeldahl method [24].

The second portion, of around 30 g, was vacuum-packed without grinding and frozen at −20 °C until the determination of the enzymatic activity of the cathepsin B and protein content.

### 2.4. Extraction of Cathepsin B from Biceps Femoris

The *biceps femoris* sample was shredded with a scalpel, removing the fat and connective tissue, and 300 mg were suspended in 3 mL extraction buffer comprising 50 mM Na citrate, 1 mM Na2-EDTA, 0.2% Triton x-100 with pH 5.0 [18,20,25,26]. The solution was homogenized twice for 20 s with Ultra-Turrax^®^ T25 Digital, the first time at 16,000 rpm and the second at 13,000 rpm. The homogenized solution was then centrifuged for 20 min, 11,000× *g* at 4 °C, and the precipitate discarded, while the supernatant liquid was used to measure both cathepsin B activity and protein content in accordance with the method reported in [27].

### 2.5. Quantification of Enzymatic Activity

The cathepsin B activity was determined by means of fluorescence measurements, using Z-Arg.Arg-7-amido-4-merhylcoumarin hydrochloride (Z-RR-AMC; Sigma-Aldrich S.r.l., Milan, Italy) as a specific substrate to avoid interference from the other cathepsins contained in the extracts [13,28]. The measurement was performed using a microplate spectrofluorometer (Molecular Devices, Spectra max Gemini xs; Molecular Devices Inc., Sunnyvale, CA, USA) with emission and excitation of 355 nm and 460 nm, respectively. In each sample containing 2.5 µM Z-RR-AMC and 50 mM sodium phosphate buffer pH 6.0, 4 mM EDTA, 2 mM DTT and 3.4 mL/L Brij 35 in 200 µL [18], 0.2–1.6 mU of cathepsin B (Sigma-Aldrich S.r.l., Milan, Italy) or 20 μL of extract were added. The kinetics were monitored for 30 min, with recording of the fluorescence every 30 s, at 37 °C. The cathepsin B activity was calculated as micromoles of AMC released per minute at 37 °C, thus expressing the activity in U/g protein or in U/g wet muscle.

### 2.6. Statistical Analyses

The statistical analysis of the data was performed by using the SPSS package, version 17 (SPSS Inc., Chicago, IL, USA). The carcass and fresh meat data were submitted to a two-way variance design, considering the three-level ‘type of dry-cured Italian PDO ham’ and the five-level ‘ripening phase’ factors.

The physical, chemical and biochemical ham characteristics were individually analyzed using the General Linear Model. First of all, a multiple covariance analysis was performed for sixty independent hams cured for a different period of days (days of processing, D), where the individual hams represent three categories (PA, SD and TO) of the factor ‘type of dry-cured Italian PDO ham’ (H_i_, with i = 1…3), in accordance with the following model:

Y_ij_ = µ + H_i_ + b_1i_ D_ij_ + b_2i_ D_ij_^2^ + b_3i_ D_ij_^3^ + b_4i_ D_ij_^4^ + ε_ij_

where Y_ij_ is a physical, chemical, or biochemical characteristic measured on the ham j (where j = 1…20) within the PDO ham type i at D_ij_ days of processing. The value µ is the mean term and ε_ij_ is the error term due to the ham j within the PDO ham type i. For this analysis, the covariates were added one at a time in sequential linear models, using the forward selection method for testing the significance of adding the new covariate to the previous model. The selected models were represented graphically. The purpose of the multiple covariance analysis was to verify, in the selected model, whether the covariance coefficients (b_ki_, with k = 1…4) within the PDO ham type differed significantly from one another, i.e., to investigate whether the multiple regression line of the physical, chemical or biochemical ham characteristics on the respective day of processing were significantly different for PA, SD, and TO. If the covariance coefficients differed significantly, a one-way variance analysis was performed for the relevant characteristic, independently for each processing phase, to compare the PDO ham types in the ‘salting’, ‘resting’, ‘drying’, ‘greasing’, or ‘curing’ phases.

After examining the sources of individual variability of each physical, chemical, and biochemical ham characteristic, the degree of their association was evaluated. The strength of the linear relationship between each pair of quantitative variables was measured by using the Pearson correlation coefficients.

## 3. Results

The initial weight of the thighs, measured immediately after dressing (Table 1), was higher for the San Daniele than for the Toscano (*p* < 0.05), with the Parma having an intermediate value.

**Figure 1:**
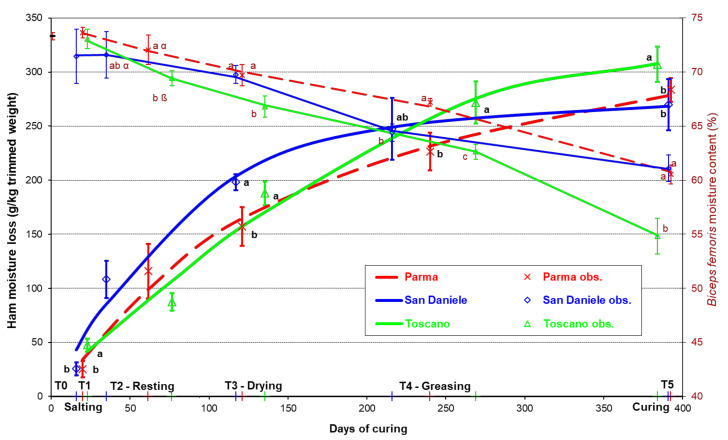
Evolution of the moisture loss in the whole ham and of the moisture content in the *biceps femoris* muscle during the processing of the main Italian prosciutto PDOs (obs. = observed mean with standard deviation values). Along the *x*–axis, the days from slaughtering of the various ripening phases of the three PDOs ham are also reported (a, b, c: differences between PDOs within a ripening phase *p* ≤ 0.05; α,β: differences between PDOs within the ripening phase *p* ≤ 0.10).

Figure 1 shows the trend of the estimated water losses from the hams and the trend of the water content of the *biceps femoris* muscle during processing, together with the days from slaughtering of the various ripening phases. The change in the water losses during maturation was significantly different in the three PDOs. The SD prosciutto, after undergoing the greatest losses in the initial processing phases, slowed this trend from T4, reaching an average value of 270 g/kg (STD 23.7 g/kg) initial weight at the end of maturation. This value was statistically no different from that of the PA prosciutto (284 g/kg; STD 10.9 g/kg) which, vice-versa, lost moisture more homogeneously during processing. The TO prosciutto presented a similar trend to the PA during the cold processing phases until drying (T3), when it underwent an acceleration of dehydration during curing in comparison with the other two types, so that at the end of maturation it was the product with the highest water losses (on average 309 g/kg initial weight; STD 16.3 g/kg; *p* < 0.05).

The moisture content of the *biceps femoris* progressively diminished from the initial value of 73% (STD 0.3%) in the raw thigh to approximately 55–60% in the cured ham, with a trend that was statistically different in the three PDOs.

**Figure 2:**
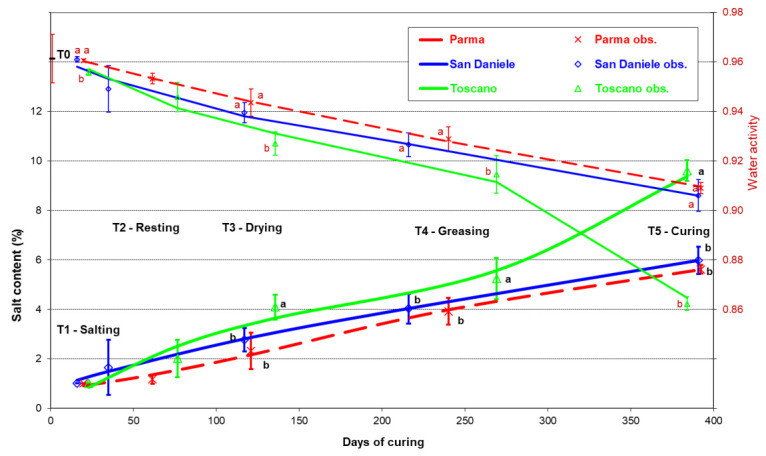
Evolution of the salt content and a_w_ in the *biceps femoris* muscle during the processing of the main Italian prosciutto PDOs (obs. = observed mean with standard deviation values; a, b: differences between PDOs within the ripening phase *p* ≤ 0.05).

Figure 2 shows the change in the water activity and salt content in the three types of prosciutto. In the TO, the water activity was significantly and increasingly lower than in the other two PDOs, which presented similar, mainly linear decreasing trends. However, the a_w_ values were slightly but steadily higher for the PA than for the SD, which reached values of around 0.90 at the end of maturation, higher than those of TO (0.86; STD 0.003; *p* < 0.05). The change in the salt content was also different in the three PDOs; at the end of processing program, TO had a sodium chloride content that was 1.5 times higher than PA and SD, which presenting values around 5.8%.

**Figure 3:**
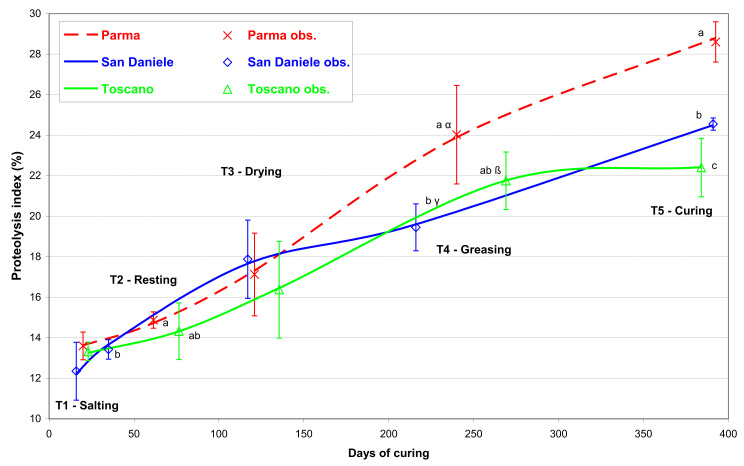
Evolution of the proteolysis index in the *biceps femoris* muscle during the processing of the main Italian prosciutto PDOs (obs. = observed mean with standard deviation values; a, b, c: differences between PDOs within a ripening phase *p* ≤ 0.05; α, β, γ: differences between PDOs within the ripening phase *p* ≤ 0.10).

Figure 3 shows the different change in proteolysis index during the PDO ham processing. It is interesting to note that from sugnatura, the PA presented a significantly higher proteolysis than the other two PDOs. At the end of ripening, the proteolysis index reached the values of 28.6% (STD 0.99%), 24.6% (STD 0.33%), and 22.4% (STD 1.44%; *p* < 0.05) in the PA, SD, and TO prosciutto, respectively.

**Figure 4:**
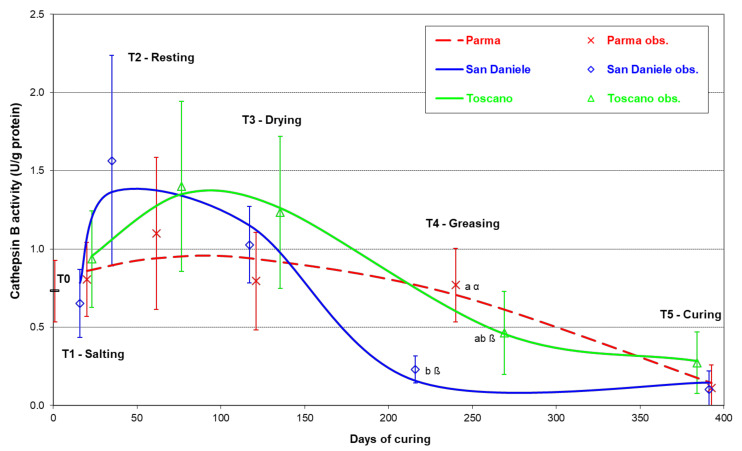
Evolution of the Cathepsin B activity in the *biceps femoris* muscle during the processing of the main Italian prosciutto PDOs (obs. = observed mean with standard deviation values; a, b: differences between PDOs within a ripening phase *p* ≤ 0.05; α, β: differences between PDOs within the ripening phase *p* ≤ 0.10).

Figure 4 shows the temporal trend of the cathepsin B activity. At T0 (which is equal for all the PDOs, as it corresponds to the fresh thighs), the average activity was 0.73 U/g protein (STD 0.196 U/g), which corresponds to 0.057 U/g wet weight (STD 0.0133 U/g). The cathepsin B activity trend was significantly influenced by the PDO type, i.e., by the different processing conditions used to produce the three studied prosciuttos. At the end of salting (T1), no differences were noted in comparison with T0 in any of the PDOs. In T2, all three PDOs showed increased activity in comparison with T0; this was especially marked in the SD and the TO. However, the differences between the three PDOs were not statistically significant due to the huge variability of the data. In the drying phase (T3), however, a considerable decrease in activity could be noted in comparison with T2 in all three PDOs, chiefly in the SD and the TO. This marked decreasing trend continued for the SD and TO hams during the first curing period, until sugnatura. In T4, the cathepsin B activity in the three PDOs differed significantly. The SD presented the lowest activity (0.23 U/g protein; STD 0.087 U/g), which correlates well with the relatively low level of proteolysis that characterized this prosciutto in T4 (Figure 3).

Finally, in T5, at the end of ripening, all of the cured hams showed a distinctly lower activity in comparison with that originally measured in the fresh meat (T0), and were not dissimilar to one another.

To obtain a more comprehensive insight into the significance of the results, the associations among the ham characteristics during processing were examined. A correlation matrix is reported in Table 2 in order to provide more details on the effects of the parameters on one another. The technological parameters were highly and linearly correlated with one another and significantly linked to the enzymatic activity.

**Figure 5:**
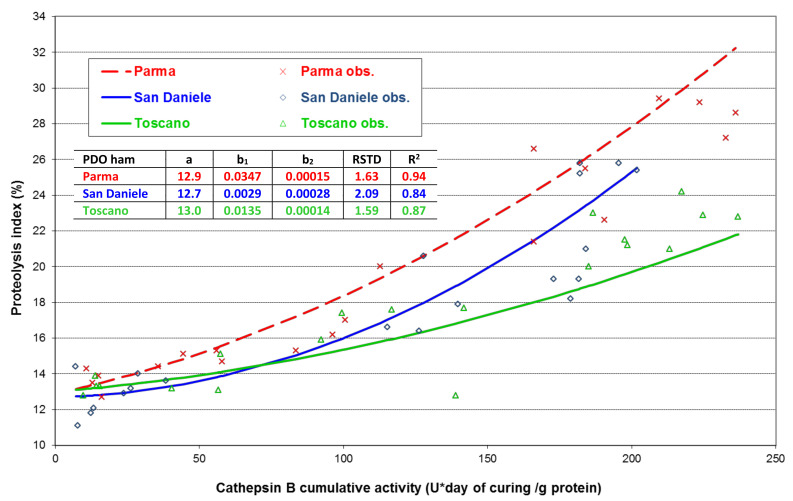
Relationship between proteolysis index (PI) and Cathepsin B cumulative activity (CBc) of Parma, San Daniele and Toscano PDO hams measured at five processing phases: individual observed values, obs., and second order polynomial regression lines, PI = a + b_1_ CBc + b_2_ CBc^2^. In table, regression coefficients (a, b_1_ and b_2_), residual standard deviation (RSTD), and coefficient of determination (R^2^) of each PDO curve are reported.

To better understand the role of cathepsin B, its cumulative activity was calculated as the definite integral at the various processing stages of the PDO trends depicted in Figure 4. The proteolysis index was then regressed against the cumulative cathepsin B activity as shown in Figure 5. The curves showed a comparable intersection value (average proteolysis index equal to 12.9% at zero cathepsin B activity). However, as the cumulative activity of the cathepsin B increased, the proteolysis increased, but at a different rate in the various PDOs, as established by a parallelism test (*p* ≤ 0.05). With an accelerated speed, at any level of cathepsin B activity, the proteolysis was greater in PA than in the saltier and drier TO hams. The SA’s trend was intermediate, but more similar to the PA hams.

## 4. Discussion

The thighs processed in accordance with the three different PDO techniques were derived from heavy pigs belonging to the same genetic type, bred and slaughtered in the same conditions, and which were homogeneous in their carcass and meat quality [21,22,23]. The degree of green thigh weight variability within the PDO is comparable with that observed in other experiments carried out in normal commercial conditions [29,30]. The weight differences between PDOs depend on the dressing procedures, which involve more extensive *semimembranosus* skinning in TO prosciutto and the retention of the trotter in SD prosciutto.

The initial trend of SD water losses can be attributed to pressing and dry salting ad libitum. In addition, the SD was characterized by a shorter pre-resting period (interval T1–T2 in Figure 1, 3 weeks in the SD vs. 6 weeks in the PA and TO), so that it enters the resting rooms—typified by a lower relative air humidity (RH: 70–80% instead of 80–85%)—earlier than the other two PDOs. The final trend, however, can be explained by the application for aesthetic reasons of the stucco. The greater expansion of the exposed muscle surface encouraged the greater final dehydration of the TO prosciutto.

Among the main thigh muscles, the *biceps femoris*, owing to its internal position, presents a higher residual moisture content [6] and a lower rate of salt diffusion [31,32] than the exposed muscles and the whole thigh. In accordance with the overall moisture loss, at the end of the curing period, the TO *biceps femoris* showed a lower moisture content (54.8%; *p* < 0.05) than the PA and SD, which had similar values (60.5 and 61.1%).

As expected [2], the different processing conditions of the three Italian PDOs had a significant influence on the final physical-chemical characteristics of the prosciutto. The change in the salt content in the three PDOs was symmetrical in comparison with that of a_w_, confirming the role of salt in immobilizing the bulk water within the tissues [31]. The TO emerge as the ham that underwent the greatest losses of moisture and a_w_, and featured the highest content of NaCl in comparison with the other two. These data are consistent with a production process which, entailing more intense trimming and increased salting, favors greater water loss and more intense assimilation of salt and, consequently, allows more bulk water to be bound. At the end of curing, the SD and PA prosciutto were characterized by comparable values of *biceps femoris* a_w_, moisture, and salt content. However, these results derived from a different evolution of water losses during ripening, which were earlier in the SD than the PA.

During dry-cured ham processing, an intense proteolysis occurs that mainly involves calpain, cathepsin, dipeptidyl peptidase, and aminopeptidase [2,6,26]. Cathepsin B was selected as the key proteinase effecting proteolysis and the evolution of its activity used for comparing the main Italian PDOs through their processing procedures [17]. In comparison with other muscles, internal muscles such as *biceps femoris* present a higher enzyme activity for longer periods of time and, thus, increased proteolysis [6].

The value of cathepsin B activity in the fresh thighs (T0) falls within the range of activity recorded in previous research, which is, however, very broad [11,13,20,25,33]. The highest values in previous studies—around 1 U/g wet weight—were obtained, in some cases, by treating the samples in such a way as to separate cathepsin B from its natural inhibitor. Comparing this value with our data, one could therefore assume that in the fresh meat, where the lysosomal structures are now damaged, a high percentage of enzyme is bound to its inhibitor [20].

The wide observed levels of cathepsin B activity variability are in line with those reported in previous studies that considered a comparable number of hams [13,18,34,35]. In this regard, it should be remembered that the enzymatic activity was measured in commercial products, controlled through the homogeneity of the raw material, but processed in ordinary production plants. A comparable trend, ascending in the first, salting and ripening, phases and descending in the successive phases, had already been shown, although it was not correlated with the processing phases [11,36], and it may be attributable to the progressive dehydration and decrease in the a_w_ of the muscle. It can be hypothesized that, after the clear increase in activity in T2, most probably due to cystatin release, this behavior was a consequence of the denaturation of the unbound enzyme, which is particularly susceptible to destabilization [37]. Such denaturation may in turn be attributed to the relatively high rate of the dehydration process observed for SD during resting, and to the variation in temperature between the resting and curing stages. On the other hand, PA showed the highest activity (0.77 U/g protein), demonstrating that the processing of this PDO ham, characterized by a lower moisture loss, inactivated the proteolytic enzyme less noticeably. In accordance with these data, the PA exhibited a significantly higher proteolysis level in T4 (Figure 3), suggesting that cathepsin B plays an essential role in this maturation phase. The TO demonstrated intermediate activity, between the PA and the SD (0.46 U/g protein), which was in agreement with its intermediate level of proteolysis. In any case, further studies are necessary to define the molecular mechanisms responsible for the different behavior in the three PDOs. It is interesting to note that the variation of activity from the start of processing to the end of ripening is comparable to that found in Spanish cured hams [38]. At this time, however, the proteolysis values differed among PDO types. PA featured the highest values, which was probably because these were cumulative and a consequence of the high level of cathepsin B activity in T4.

The process of water loss from the whole ham during seasoning was determined and accompanied by parallel changes in the *biceps femoris* parameters, such as the reduction in moisture content and a_w_ value and an increase of salt penetration and content. In its turn, the cathepsin B activity was negatively correlated with moisture loss and salt concentration and positively associated with moisture content and a_w_, which is in agreement with the relationship reported in previous research [18,39]. The proteolysis index, which is a cumulative variable that increases during seasoning due to the successive addition of the hydrolysis products, was correlated with the technological parameters in a direction determined by the specific trends they each underwent during ripening.

The comparable intersection value of the different PDO types in Figure 5 confirms the homogeneity of the fresh thighs. Moreover, this result indicates that muscle protein degradation was initially driven by enzymes other than cathepsin B, particularly calpains [34], that take part in the early breakdown of myofibrillar proteins during the salting and early post-salting stages [6]. Starting in the salting and resting phases, the role of cathepsin B became important and remained active throughout the whole curing process [13]. Different percentages of potential cathepsin B activity, measured in the assay under optimal conditions, were effective on the muscle proteins during ripening in the three PDOs. A discrepancy between the “potential” and “actual” cathepsin B activity has been reported in Jinhua ham and assigned to the effect of the technological conditions, such as temperature, salt content, and pH, on enzyme activity [18]. This might also have been the case in our study, given that salt content strongly inhibited cathepsin B activity [18] and that we observed the lowest proteolysis index in the saltier TO ham. However, the addition of pepper and seasonings could also have further inhibited the enzyme activity during TO ripening [40]. Moreover, an additional contribution related to the differential influence of the three technological conditions on the muscle protein accessibility to the endoprotease cannot be excluded, given that high salt also favors protein aggregation [41].

The different technological conditions and intensity of proteolysis produced specific sensory properties in the various PDO hams, as demonstrated in the profile provided by trained assessors and confirmed by consumer preferences. Indeed, the hams were subjected to sensory analysis, both descriptive and hedonistic, the results of which are being published elsewhere [21,22]. Briefly, the panelists perceived the TO hams as redder, saltier, harder, more fibrous and drier than the PA and SD products [21,22]. This outcome agrees with those of other authors [19,42], who showed that hardness decreases with increasing proteolysis. Furthermore, the TO was characterized by a greater intensity of pork flavor, as well as a more rancid flavor. The SD and PA hams were perceived as sweeter and with less ageing flavor than the TO [21,22]. The PDO type also influenced consumer satisfaction, the appreciation scores for the PA and SD differing from those for the TO, and associated with quantitative descriptive analysis results according to consumer origin [22]. However, sensory defects, such as bitter taste [35], mushy texture, and pastiness [16,43] detected in highly proteolyzed PA hams, were not perceived. This is likely to have been because cathepsin B did not reach the required high thresholds of activity in the green thighs.

## 5. Conclusions

The hams produced from homogeneous green thighs through the processing procedures of the main Italian PDOs, Parma, San Daniele, and Toscano, revealed specific technological and biochemical characteristics, likely resulting in the peculiar sensory properties highlighted in the parallel studies carried out on the same PDOs, and clearly perceived by consumers.

Within this framework, the trend of cathepsin B activity during the ripening of the various PDOs was significantly different, particularly in the intermediate curing phases. This activity correlates with the proteolysis index through a PDO-dependent pattern. Indeed, at the same cumulative level of cathepsin B activity, the saltiest and driest TO ham, the only ham with pepper and seasonings added, differed from the others by a lower proteolysis index. Then again, in the PA ham, which is known to be more prone to defects related to excess proteolysis, the degradation effect on the muscular proteins was the highest, suggesting the existence of conditions contributing to a higher “actual” cathepsin B activity during ripening.

## Figures and Tables

**Table 1:** Weight of green thighs which produced the experimental hams, averaged as prosciutto PDO type marginal means.

Item		Prosciutto PDO Type			RSTD	Significance		
		Parma	San Daniele	Toscano		PDO	Ripening Phase (T)	PDOxT
Initial thigh weight	kg	14.20 ^a,b^	14.43 ^a^	13.59 ^b^	0.993	0.03	0.15	0.78

^a,b^ Means with different upper letter differ significantly (*p* ≤ 0.05).

**Table 2:** Bivariate coefficient of correlation between ham characteristics (measured on *biceps femoris* if not otherwise specified; no. of hams = 60).

	Moisture Loss of Ham	Moisture Content	a_w_	Salt Content	Proteolysis Index
**Cathepsin B activity**	−0.485	0.586	0.522	−0.541	−0.580
**Moisture loss of ham**		−0.867	−0.870	0.868	0.851
**Moisture content**			0.950	−0.949	−0.771
**a_w_**				−0.990	−0.741
**Salt content**					0.754

All the reported coefficients are significant (*p* ≤ 0.01).

## References

[1] RIFT-Registro Italiano Filiera Tutelata. https://www.csqa.it/CSQA/Download/PROSCIUTTO-DI-PARMA-DOP.

[2] Toldrá F. (2006). The role of muscle enzymes in dry-cured meat products with different drying conditions. Trends Food. Sci. Technol..

[3] Toldrá F. (2002). Manufacturing of dry-cured ham. Dry-Cured Meat Products.

[4] Pugliese C., Sirtori F., Skrlep M., Piasentier E., Calamai L., Franci O., Candek-Potokar M. (2015). The effect of ripening time on the chemical, textural, volatile and sensorial traits of Bicep femoris and Semimembranosus muscles of the Slovenian dry-cured ham Kraški pršut. Meat Sci..

[5] Wu W., Zhou Y., Wang G., Zhu R., Ge C., Liao G. (2020). Changes in the physicochemical properties and volatile flavor compounds of dry-cured Chinese Laowo ham during processing. J. Food Process. Preserv..

[6] Toldrá F., Flores M., Sanz Y. (1997). Dry-cured ham flavour: Enzymatic generation ad process influence. Food Chem..

[7] Goll D.E., Neti G., Mares S.W., Thompson V.F. (2008). Myofibrillar protein turnover: The proteasome and the calpains. J. Anim. Sci..

[8] Söderstroöm M., Salminen H., Glumoff V., Kirschke H., Aro H., Vuorio E. (1999). Cathepsin expression during skeletal development. Biochim. Biophys. Acta.

[9] Turk B., Turk D., Turk V. (2000). Lysosomal cysteine proteases: More than scavengers. Biochim. Biophys. Acta.

[10] Bechet D., Tassa A., Taillandier D., Combaret L., Attaix D. (2005). Lysosomal proteolysis in skeletal muscle. Int. J. Biochem. Cell Biol..

[11] Conti V., Ramoni R., Parolari G., Virgili R., Grolli S., Accornero P., Fermi P., Biffi R., Bignetti E. (1997). Evalution of cathepsin B levels in fresh thighs selected for cured raw ham product. Meat Sci..

[12] Riccio M., Di Giaimo R., Pianetti S., Palmieri P., Melli M., Santi S. (2001). Nuclear Localization of Cystatin B, the Cathepsin Inhibitor Implicated in Myoclonus Epilepsy (EPM1). Exp. Cell Res..

[13] Larrea V., Hernando I., Quiles A., Lluch M.A., Pérez-Munuera I. (2006). Changes in proteins during Teruel dry-cured ham processing. Meat Sci..

[14] Baron P.C., Jacobsen S., Purslow P. (2004). Cleavage of desmin by cysteine proteases: Calpains and cathepsin B. Meat Sci..

[15] Schivazappa C., Degni M., Nanni Costa L., Russo V., Buttazzoni L., Virgili R. (2002). Analysis of raw meat to predict proteolysis in PA ham. Meat Sci..

[16] Škrlep M., Čandek-Potokar M., Mandelc S., Javornik B., Gou P., Chambon C., Santé-Lhoutellier V. (2011). Proteomic profile of dry-cured ham relative to PRKAG3 or CAST genotype, level of salt and pastiness. Meat Sci..

[17] Zhou C.Y., Pan D.D., Cao J.X., Zhou G.H. (2021). A comprehensive review on molecular mechanism of defective dry-cured ham with excessive pastiness, adhesiveness, and bitterness by proteomics insights. Compr. Rev. Food Sci. Food Saf..

[18] Zhao G.M., Zhou G.H., Wang Y.L., Xu X.L., Huan Y.J., Wu J.Q. (2005). Time-related changes in cathepsin B and L activities during processing of Jinhua ham as a function of pH, salt and temperature. Meat Sci..

[19] Parolari G., Virgili R., Schivazappa C. (1994). Relationship between cathepsin B activity and compositional parameters in dry-cured hams of normal and defective texture. Meat Sci..

[20] García-Garrido J.A., Quiles-Zafra R., Tapiador J., Luque de Castro M.D. (2000). Activity of cathepsin B, D, H and L in Spanish dry-cured ham of normal and defective texture. Meat Sci..

[21] Laureati M., Buratti S., Giovanelli G., Corazzin M., Lo Fiego D.P., Pagliarini E. (2014). Characterization and differentiation of Italian Parma, San Daniele and Toscano dry-cured hams: A multi-disciplinary approach. Meat Sci..

[22] Pagliarini E., Laureati M., Dinnella C., Monteleone E., Proserpio C., Piasentier E. (2016). Influence of pig genetic type on sensory properties and consumer acceptance of Parma, San Daniele and Toscano dry-cured hams. J. Sci. Food Agric..

[23] Fabbro A., Bencivenni M., Piasentier E., Sforza S., Stecchini M.L., Lippe G. (2016). Proteolytic resistance of actin but not of myosin heavy chain during processing of Italian PDO (protected designation of origin) dry-cured hams. Eur. Food Res. Technol..

[24] AOAC (2016). Official Methods of Analysis, of AOAC International.

[25] Sturaro E., Gallo L., Noventa M., Carnier P. (2008). The genetic relationship between enzymatic activity of cathepsin B and firmness of dry-cured hams. Meat Sci..

[26] Toldrá F., Flores M. (2000). The use of muscle enzymes as predictors of pork meat quality. Food Chem..

[27] Lowry O.H., Rosebrough N.J., Farr A.L., Randall R.J. (1951). Protein measurement with the Folin phenol reagent. J. Biol. Chem..

[28] Armero E., Barbosa J., Toldrá F., Baselga M., Pla M. (1999). Effects of the terminal sire type and sex on pork muscle cathepsins (B, B+L and H), cysteine proteinase inhibitors and lipolytic enzyme. Meat Sci..

[29] Renaville B., Piasentier E., Fan B., Vitale M., Prandi A., Rothschild M.F. (2010). Candidate gene markers involved in San Daniele ham quality. Meat Sci..

[30] Vitale M., Corazzin M., Favotto S., Saccà E., Piasentier E. (2009). Variability in the characteristics of fresh meat and thighs in relationship to genetic type of the heavy pig. Ital. J. Anim. Sci..

[31] Monin G., Marinava P., Talmant A., Martin J.F., Cornet M., Lanore D., Grasso F. (1997). Chemical and Structural changes in Dry-cured ham, (Bayonne hams) during processing and effects of the dehairing technique. Meat Sci..

[32] Innocente N., Manzocco L., Nicoli M.C., Piasentier E. (2009). Monitoring moisture distribution in San Daniele ham by magnetic resonance imaging. Qualità e Sicurezza Nella Filiera del Prosciutto [Quality and Safety in Prosciutto Chain].

[33] Bechet D., Deval C., Robelin J., Ferrara M., Obled A. (1996). Developmental Control of Cathepsin B Expression in Bovine Fetal Muscles. Arch. Biochem. Biophys..

[34] Sárraga C., Gil M., García-Regueiro J.A. (1993). Comparison of Calpain and Cathepsin (B, L e D) Activities during Dry-Cured Ham Processing from Heavy and Light Large White Pigs. J. Sci. Food Agric..

[35] Sforza S., Piazzani A., Motti M., Porta C., Virgili R., Galaverna G., Dossena A., Marchelli R. (2001). Oligopeptides and free amino acids in Parma hams of known cathepsin B activity. Food Chem..

[36] Takahiko A., Masaki Y., Ryuji U. (2002). A cathepsin B-like enzyme from mackerel white muscle is a precursor of cathepsin B. Comp. Biochem. Physiol. B Biochem. Mol. Biol..

[37] Mort J., Buttle D. (1997). Molecules in focus: Cathepsin B. Int. J. Biochem. Cell Biol..

[38] Toldrá F., Rico E., Flores J. (1993). Cathepsins B, D, H and L activity in the processing of dry-cured ham. J. Sci. Food Agric..

[39] Rico E., Toldrà F., Flores M. (1991). Effect of dry-curing process parameters on pork muscle cathepsin B, H and L. Eur. Food Res. Technol..

[40] Munekata P.E.S., Rocchetti G., Pateiro M., Lucini L., Domínguez R., Lorenzo J.M. (2020). Addition of plant extracts to meat and meat products to extend shelf-life and health-promoting attributes: An overview. Curr. Opin. Food Sci..

[41] Martin L., Cordoba J.J., Antequera T., Timón M., Ventanas J. (1998). Effects of salt and temperature on proteolysis during ripening of Iberian ham. Meat Sci..

[42] Ruiz-Ramírez J., Arnau J., Serra X., Gou P. (2006). Effect of pH24, NaCl content and proteolysis index on the relationship between water content and texture parameters in biceps femoris and semimembranosus muscles in dry-cured ham. Meat Sci..

[43] Virgili R., Parolari G., Schivazappa C., Bordini C.S., Borri M. (1995). Sensory and texture quality of dry-cured ham as affected by endogenous cathepsin B activity and muscle composition. J. Food Sci..

